# Citrate- vs. acetate-based dialysate in bicarbonate haemodialysis: consequences on haemodynamics, coagulation, acid-base status, and electrolytes

**DOI:** 10.1186/1471-2369-10-7

**Published:** 2009-03-05

**Authors:** Luca Gabutti, Barbara Lucchini, Claudio Marone, Lorenzo Alberio, Michel Burnier

**Affiliations:** 1 Division of Nephrology, Ospedale la Carità, Via Ospedale, 6600 Locarno, Switzerland; 2 Department of Internal Medicine, Ospedale la Carità, Locarno, Switzerland; 3 Department of Internal Medicine, Ospedale San Giovanni, Bellinzona, Switzerland; 4 Department of Hematology, University Hospital of Bern, Bern, Switzerland; 5 Division of Nephrology, University Hospital of Lausanne, Lausanne, Switzerland

## Abstract

**Background:**

A concentrate for bicarbonate haemodialysis acidified with citrate instead of acetate has been marketed in recent years. The small amount of citrate used (one-fifth of the concentration adopted in regional anticoagulation) protects against intradialyser clotting while minimally affecting the calcium concentration. The aim of this study was to compare the impact of citrate- and acetate-based dialysates on systemic haemodynamics, coagulation, acid-base status, calcium balance and dialysis efficiency.

**Methods:**

In 25 patients who underwent a total of 375 dialysis sessions, an acetate dialysate (A) was compared with a citrate dialysate with (C+) or without (C) calcium supplementation (0.25 mmol/L) in a randomised single-blind cross-over study. Systemic haemodynamics were evaluated using pulse-wave analysis. Coagulation, acid-base status, calcium balance and dialysis efficiency were assessed using standard biochemical markers.

**Results:**

Patients receiving the citrate dialysate had significantly lower systolic blood pressure (BP) (-4.3 mmHg, p < 0.01) and peripheral resistances (PR) (-51 dyne.sec.cm^-5^, p < 0.001) while stroke volume was not increased. In hypertensive patients there was a substantial reduction in BP (-7.8 mmHg, p < 0.01). With the C+ dialysate the BP gap was less pronounced but the reduction in PR was even greater (-226 dyne.sec.cm^-5^, p < 0.001). Analyses of the fluctuations in PR and of subjective tolerance suggested improved haemodynamic stability with the citrate dialysate. Furthermore, an increase in pre-dialysis bicarbonate and a decrease in pre-dialysis BUN, post-dialysis phosphate and ionised calcium were noted. Systemic coagulation activation was not influenced by citrate.

**Conclusion:**

The positive impact on dialysis efficiency, acid-base status and haemodynamics, as well as the subjective tolerance, together indicate that citrate dialysate can significantly contribute to improving haemodialysis in selected patients.

**Trial registration:**

ClinicalTrials.gov NCT00718289

## Background

A concentrate acidified with citric acid instead of the less physiologic acetic acid has been successfully implemented in the United States for bicarbonate haemodialysis over the past 7 years [1-3]. In contrast to traditional regional citrate anticoagulation, the small amount of citrate used in the acid concentrate (0.8 mmol/L; only about one-fifth of the concentration necessary to achieve anticoagulation [1,4,5]) affects the calcium concentration and the locally enhanced coagulation activation in a limited way, resulting in approximately 10% reduction in post-dialysis ionized calcium and in no measurable systemic anticoagulation [1]. The absence of significant systemic repercussions is related not only to the low amount of citrate used but also to the rapid conversion of citrate into bicarbonate, which takes place in the liver and muscles and results in a higher post-dialysis bicarbonataemia [1,6,7]. Despite the rapid clearance of citrate, the local consequences of removing calcium from the blood clotting cascade have measurable positive effects on the dialyser life-span in the "reuse" modality and on dialysis quality, as quantified by urea Kt/V [1,3]. The improvement in urea clearance has been correlated with an assumed favourable effect on dialyser fibre permeability mediated by the intradialyser anticoagulant properties of citrate [1,3,8]. Considering the importance of limiting the biocompatibility-related coagulation activation taking place in the extracorporeal circuit [9-17], the availability of a simple way to inhibit it without affecting systemic coagulation and bleeding risk [18] is very promising.

Although thousands of patients have been treated in recent years with haemodialysis fluids based on citric instead of acetic acid, the haemodynamic tolerance (the reduction in ionized calcium concentration and the increase in bicarbonateamia could both result in a lower intra-dialytic blood pressure [19-25]) and the amount of systemic coagulation activation related to each one of the modalities, have not been investigated.

The aim of this randomised, controlled, single-blind, cross-over study in single-use dialyser bicarbonate haemodialysis was to detail the consequences on systemic haemodynamics (primary outcome) and on coagulation activation, acid-base status, calcium balance and dialysis efficiency (secondary outcomes) of using citric instead of acetic acid in haemodialysis fluids.

## Methods

**Figure 1:**
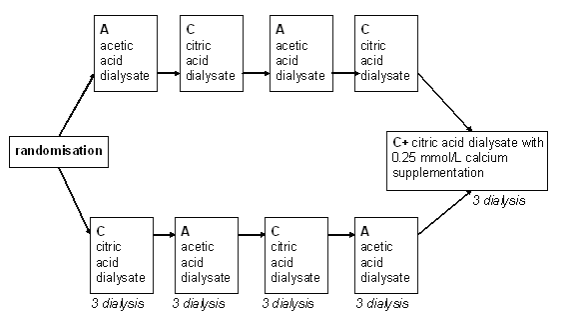
**Study design**. Schematic representation of the study design.

Twenty-five chronic haemodialysis patients (15 male and 10 female) (sample size arbitrarily set in the absence of previous data or studies with an analogous primary outcome), undergoing dialysis in the dialysis unit of the Ospedale la Carità (Locarno, Switzerland) 3 to 4 hours three times a week who were clinically stable and without intercurrent illnesses were enrolled in the study. A single-blind, cross-over design was used in which the patients were initially randomised (one to one beginning with acetic acid dialysate according to the enrollement sequence) into one of two arms of the study i.e. receiving either acetic acid (modality A) or citric acid (modality C) dialysate. In the following 3 weeks the modality was switched weekly to the alternative one. Finally, with the intention to compensate for the reduction in serum calcium induced by citrate binding, both study arms were completed by a week in which patients were administered a citric acid dialysate containing a calcium supplement of 0.25 mmol calcium/L (modality C+) (see Figure 1 for details).

The haemodialyses were performed using a 4008 H machine equipped with a cartridge of bicarbonate Bibag^© ^and a high-flux single-use modified polysulfone membrane (Helixone^©^; FX 80 or 100 with an effective surface area of 1.8 and 2.2 m^2 ^and an ultrafiltration coefficient of 59 and 73 ml/h mmHg, respectively), all from Fresenius Medical Care (Bad Homburg, Germany). Dialysis fluids were produced by Bichsel AG (Interlaken, Switzerland); the fluid composition was as following: sodium 138 mmol/L, magnesium 0.5 mmol/L, glucose 5.5 mmol/L, potassium 2 mmol/L (individually adapted according to the pre-dialysis serum potassium), acetate 3.0 mmol/L in A and 0.3 in C and C+, citrate 0 mmol/L in A and 0.8 in C and C+ and calcium 1.25 and 1.50 mmol/L in A and C and 1.50 and 1.75 in C+. Intradialytic heparin anticoagulation was carried out according to standard procedures and continued without dose adaptations in both arms of the study; priming of the dialyser was heparin free. The prescribed dialyser effective surface area, dialysis fluid conductibility, temperature and composition (with the exception of acetate, citrate and calcium), and effective blood flow were recorded at enrolment in the study and were left unchanged for the following 5 weeks. The medications of the patients (including phosphate binders) were also left unchanged.

Serum BUN, creatinine, potassium, phosphate, calcium and whole-blood pH, bicarbonate and ionised calcium were measured at the beginning and at the end of the third dialysis session of each week. Ionised and total calcium were also measured at the beginning and at the end of the first dialysis session of each week. Prothrombin fragments 1+2 (F1+2) and thrombin-antithrombin complexes (TAT) were determined at the beginning and at the end of the third dialysis session of each week [26]. Blood samples were taken from the arterial limb of the shunt.

Systolic and diastolic blood pressures and heart rate were measured before the start of each session and then repeated at 30-min intervals throughout dialysis with an automated Blood Pressure Monitor 4008 (Fresenius Medical Care, Bad Homburg, Germany) integrated in the dialysis machine. Stroke volumes (integrated mean of the flow waveform between the current upstroke and the dicrotic notch) and peripheral resistances (ratio of mean arterial pressure to stroke volume multiplied by heart rate) were evaluated between 5 and 10 minutes after the start of the session and then every 45 minutes using a finger beat-to-beat monitor Finometer^© ^(Finapres Medical Systems BV, Arnhem, The Netherlands). The use of isotonic saline infusions (100–200 ml) to treat symptomatic hypotension or symptoms related to intravascular hypovolaemia was registered.

Kt/V was calculated using a second-generation single-pool Daugirdas formula (Kt/V = -ln(R-0.03) + [(4-3.5 × R) × (UF/W)] where R = post-dialysis BUN/pre-dialysis BUN, UF = net ultrafiltration and W = weight).

Citrate accumulation during dialysis was estimated by calculating the calcium gap = |post – predialysis total calcium| - |post – predialysis ionised calcium|[6]. In a post-hoc analysis, 0.2 mmol/L was used as a cut-off value to classify patients as rapid (< 0.2 mmol/L) or slow (= 0.2 mmol/L) metabolisers.

**Figure 2:**
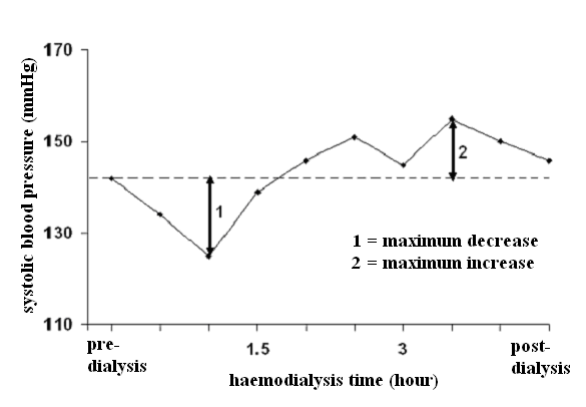
**Evaluation of the fluctuations in haemodynamic parameters**. Schematic representation of the method used to calculate the maximum increase and decrease of each measured parameter during the dialysis session.

Results were expressed as mean ± SD. Statistical analyses were performed using a statistical software package (SPSS 12.0; SPSS Inc., Chicago, IL, USA). Laboratory and haemodynamic parameters were compared first with an ANOVA and then, if significant, by a paired t-test of the mean of the values obtained in each patient with each modality. Haemodynamic parameters as a function of dialysis time were compared using a trapezoidal estimation of the area under the curves followed by a Wilcoxon signed rank test. To better understand the haemodynamic changes, an evaluation of the maximum increase and decrease in each parameter was added to the data analysis (see Figure 2 for an explanation of the calculation). Percentages were compared using Fisher's exact test. In all cases, *p *= 0.05 was considered statistically significant; *p *was expressed as ns (not significant), = 0.05, < 0.05, < 0.01 and < 0.001.

The protocol of the study was approved by the local Ethical Committee. All patients gave written informed consent prior to enrolment in the study.

## Results

### Characteristics of the studied population

The characteristics of the studied population (*n *= 25) at the time of enrollment were as follows (mean ± SD): age 71.1 ± 10.1 years, weight 74.4 ± 16.4 kg, male/female ratio 1.5. The baseline haemodialysis prescriptions were: dialyser effective surface area 1.86 ± 0.15 m^2^; dialysis duration 3.54 ± 0.43 h; dialysis fluid conductibility 13.8 ms/cm; dialysis fluid temperature 36.5°C; effective blood flow 305.64 ± 58.90 ml/min; dialysis fluid flow rate 536 ± 99.50 ml/min and dialysis fluid concentration for potassium 3.00 ± 0.75 mmol/L, magnesium 0.5 mmol/L, glucose 5.5 mmol/L and acetate 3 mmol/L. See Table 1 for details including underlying nephropathies, comorbidities and antihypertensive drugs in use. All participants completed the 5 phases of the protocol (Figure 1). The study was started on March 2007 and ended on November of the same year.

**Table 1:** Characteristics of the studied population. Characteristics of the cohort at the beginning of the study

Patient no.	Sex	Age (y)	Underlying nephropathy	Comorbidities		Medication				Dry weight (kg)	Dialyser surface area(m^2^)	Dialysis Duration (h)
							
				Ischemic cardio-myopathy	Diabetes mellitus	Beta-blockers	Calcium antagonists	Alpha-blockers	ACE-inhibitors or ARB			
1	F	64	Nephroangiosclerosis	N	N	Y	Y	N	Y	57.00	1.8	3.0
2	F	56	Nephroangiosclerosis	N	N	Y	Y	N	Y	64.00	1.8	3.5
3	F	88	Nephroangiosclerosis	N	N	Y	N	Y	Y	64.00	1.8	3.0
4	M	59	Focal segmental glomerulosclerosis	N	Y	Y	N	N	Y	80.0	2.2	3.5
5	F	75	Diabetic nephropathy	N	Y	Y	Y	Y	Y	48.50	1.8	3.5
6	M	74	Nephroangiosclerosis	N	N	N	N	N	N	71.00	1.8	4.0
7	M	68	IgA nephropathy	Y	N	N	N	N	Y	94.65	1.8	4.0
8	F	65	Diabetic nephropathy	Y	Y	Y	Y	Y	Y	47.00	1.8	3.5
9	F	66	Nephroangiosclerosis	N	N	N	Y	Y	N	49.00	1.8	3.5
10	M	77	Interstitial Nephritis	N	N	N	Y	N	Y	99.50	1.8	3.5
11	M	72	Nephroangiosclerosis	Y	N	Y	N	N	Y	92.00	1.8	3.0
12	F	48	IgA nephropathy	N	Y	Y	N	N	N	64.50	2.2	3.5
13	M	77	Nephroangiosclerosis	Y	N	Y	Y	N	N	89.40	1.8	3.0
14	M	81	Nephroangiosclerosis	N	N	Y	Y	N	N	79.65	1.8	3.5
15	M	59	Diabetic nephropathy	Y	Y	Y	N	N	Y	99.00	1.8	4.0
16	M	88	Nephroangiosclerosi	N	N	Y	N	N	N	65.15	1.8	4.0
18	M	64	Nephroangiosclerosis	N	Y	Y	Y	Y	N	97.00	1.8	3.5
17	F	74	Diabetic nephropathy	N	Y	N	Y	N	N	80.00	1.8	3.5
19	M	65	Focal segmental glomerulosclerosis	N	N	Y	N	N	N	86.00	2.2	4.0
20	F	75	Diabetic nephropathy	Y	Y	Y	N	N	Y	68.50	1.8	3.0
21	M	81	Nephroangiosclerosis	Y	N	Y	N	N	Y	69.20	1.8	4.0
22	M	72	Diabetic nephropathy	Y	Y	N	N	N	Y	88.00	1.8	4.0
23	M	88	Nephroangiosclerosis	Y	Y	N	Y	N	Y	83.50	1.8	3.0
24	M	71	Nephroangiosclerosis	Y	Y	Y	N	N	Y	74.50	2.2	4.5
25	F	70	Nephroangiosclerosis	N	N	Y	Y	N	Y	48.20	1.8	3.0

M, male; F, female; Y, yes; N, no; ACE, angiotensin-converting enzyme; ARB, angiotensin receptor antagonists

### Haemodynamic consequences of dialysis fluids containing citrate instead of acetate

**Figure 3:**
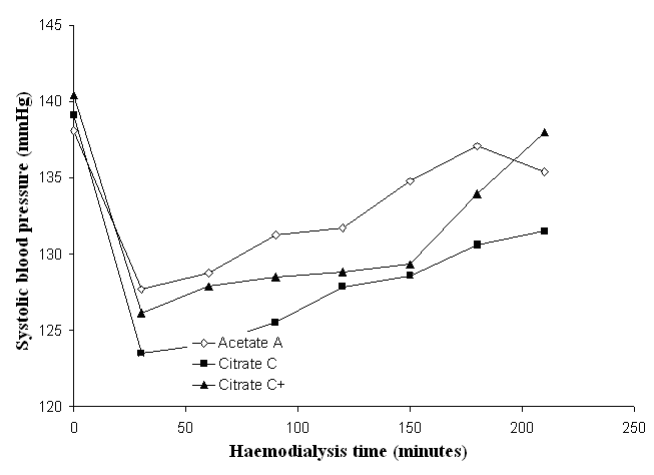
**Systolic blood pressure**. Systolic blood pressure as a function of the haemodialysis time using, respectively, acetate (A) (empty diamonds), calcium uncorrected citrate (C) (black squares) and calcium supplemented citrate dialysate (C+) (black triangles).

**Figure 4:**
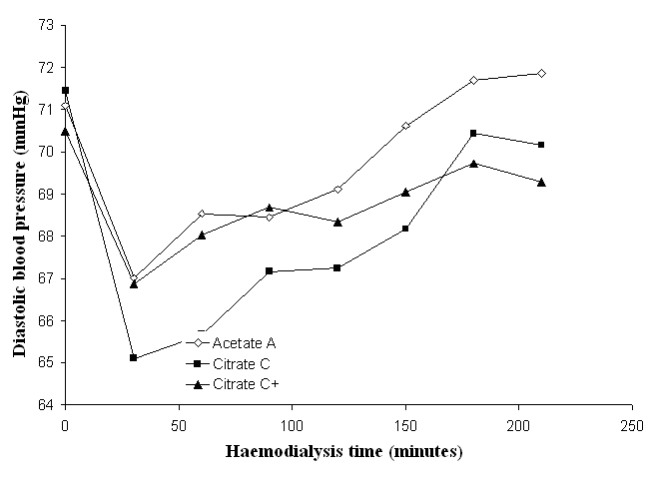
**Diastolic blood pressure**. Diastolic blood pressure as a function of the haemodialysis time using, respectively, acetate (A) (empty diamonds), calcium uncorrected citrate (C) (black squares) and calcium supplemented citrate dialysate (C+) (black triangles).

**Figure 5:**
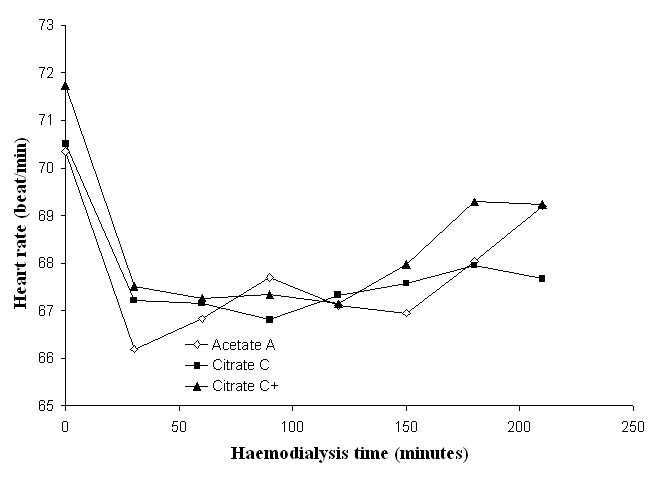
**Heart rate**. Heart rate as a function of the haemodialysis time using, respectively, acetate (A) (empty diamonds), calcium uncorrected citrate (C) (black squares) and calcium supplemented citrate dialysate (C+) (black triangles).

**Figure 6:**
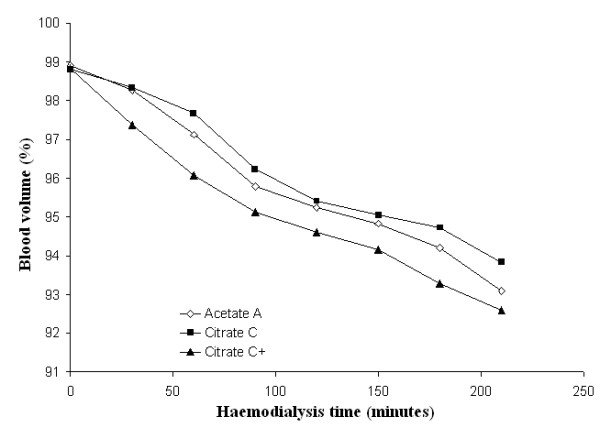
**Blood volume**. Blood volume as a function of the haemodialysis time using respectively acetate (A) (empty diamonds), calcium uncorrected citrate (C) (black squares) and calcium supplemented citrate dialysate (C+) (black triangles).

**Figure 7:**
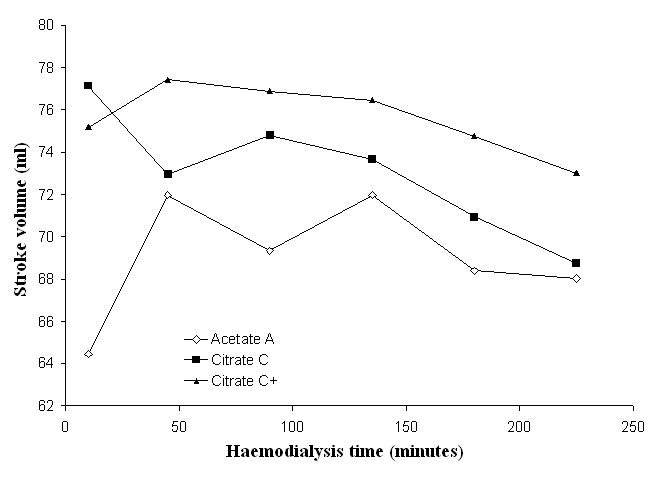
**Stroke volume**. Stroke volume as a function of the haemodialysis time using, respectively, acetate (A) (empty diamonds), calcium uncorrected citrate (C) (black squares) and calcium supplemented citrate dialysate (C+) (black triangles).

**Figure 8:**
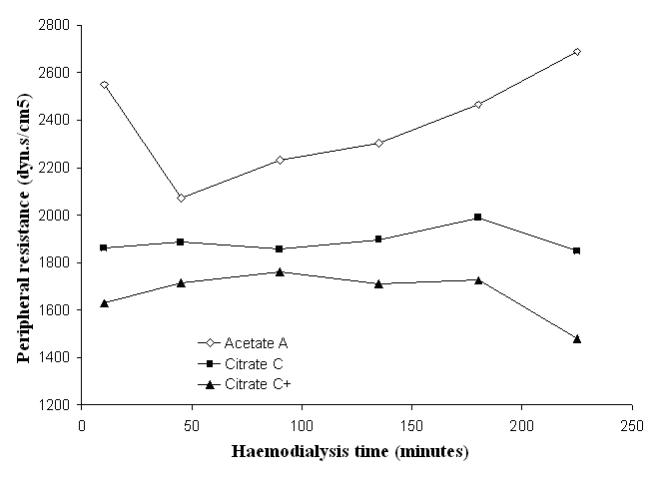
**Peripheral resistance**. Peripheral resistance as a function of the haemodialysis time using, respectively, acetate (A) (empty diamonds), calcium uncorrected citrate (C) (black squares) and calcium supplemented citrate dialysate (C+) (black triangles).

Mean systolic and diastolic blood pressures and mean peripheral resistances in patients dialysed with citrate dialysate were significantly lower than in patients dialysed with acetate (-4.3 and -1.6 mmHg and -51 dyne.sec.cm^-5^; *p *< 0.01 and *p *< 0.001 respectively). The associated slight increase in stroke volume (+5.0 ml) was not significant. Dialysate supplemented with 0.25 mmol calcium/L to compensate for binding to citrate (see the following paragraph for details) reduced the blood pressure gap between the two dialysis schedules to a nonsignificant value (-2.0 and 0.8 mmHg respectively) whereas the gap in peripheral resistances increased further (-226 dyne.sec.cm^-5^; *p *< 0.001) (see Table 2 for details). Blood pressure, heart rate, blood volume, peripheral resistances and stroke volume as a function of haemodialysis time are depicted in Figures 3, 4, 5, 6, 7, 8.

**Table 2:** Haemodynamic consequences of using citric instead of acetic acid in dialysis fluids.

	Acetate (A)	Citrate (C)	Citrate (C+)	MD (AC)	MD (AC+)	P(Avs.C)	P(Avs.C+)
**Systolic BP **(mmHg)	**132.8 **± 15.5	**128.5 **± 14.1	**130.7 **± 20.2	4.3	2.1	**< 0.01**	ns
**Diastolic BP**(mmHg)	**69.5 **± 9.8	**67.9 **± 9.1	**68.7 **± 11.6	1.6	0.8	**< 0.05**	ns
**Heart rate **(beat/min)	**67.6 **± 10.8	**67.8 **± 12.3	**68.3 **± 12.5	-0.2	-0.7	ns	ns
**Blood volume **(%)	**96.3 **± 2.8	**96.6 **± 2.9	**95.6 **± 3.1	-0.3	0.7	ns	ns
**Stroke volume **(ml)	**56.6 **± 16.1	**61.5 **± 14.8	**63.5 **± 13.1	-5.0	-6.9	ns	ns
**Peripheral resistence **(dyne.sec.cm^-5^)	**1633 **± 524	**1581 **± 410	**1407 **± 235	51	226	**< 0.001**	**< 0.001**

Blood pressure (BP) and pulse-wave analysis results based on the area under the curve using acetate dialysate (A) vs. citrate dialysate with (C+) or without (C) calcium supplementation.

ns, non significant, MD: mean difference

This observation was confirmed in an analysis of the maximum fluctuations in systolic blood pressure during dialysis, in which the citrate dialysate produced, respectively, a larger decrease and a less pronounced increase (+6.4 and -4.9 mmHg; *p *< 0.01 and *p *< 0.05 respectively).

Surprisingly, the opposite was observed regarding peripheral resistances with a less pronounced decrease (-489 dyne.sec.cm^-5^; p < 0.001) and a similar maximum increase that was confirmed with citrate dialysate supplemented with 0.25 mmol calcium/L (see Table 3 for details).

**Table 3:** Haemodynamic consequences of using citric instead of acetic acid in dialysis fluids II.

		Acetate (A)	Citrate (C)	Citrate (C+)	P(Avs.C)	P(Avs.C+)
**Systolic BP **(mmHg)	max.decrease	**19.2 **± 9.61	**25.6 **± 10.98	**23.4 **± 13.43	**< 0.01**	ns
	max.increase	**13.96 **± 8.28	**9.07 **± 7.84	**11.01 **± 8.32	**< 0.05**	ns
**Diastolic BP **(mmHg)	max.decrease	**10.60 **± 4.06	**12.18 **± 5.13	**10.68 **± 5.99	ns	ns
	max.increase	**7.42 **± 5.56	**7.33 **± 5.54	**7.95 **± 5.03	ns	ns
**Blood volume **# (%)	max.decrease	**6.05 **± 4.21	**4.81 **± 3.51	**6.47 **± 3.88	ns	ns
	max increase	**0.99 **± 0.95	**1.21 **± 1.26	**0.44 **± 0.70	ns	**< 0.05**
**Heart rate **# (beat/min)	max.decrease	**8.04 **± 5.99	**7.79 **± 5.76	**7.81 **± 6.92	ns	ns
	max increase	**4.03 **± 3.21	**3.27 **± 2.95	**4.03 **± 4.28	ns	ns
**Stroke volume **(ml)	max.decrease	**17.20 **± 9.78	**19.62 **± 10.41	**14.90 **± 16.65	ns	ns
	max.increase	**28.14 **± 15.22	**28.26 **± 32.24	**23.04 **± 23.97	ns	ns
**Peripheral resistence **	max.decrease	**973.60 **± 571.25	**485.30 **± 267.25	**410.37 **± 330.91	**< 0.001**	**< 0.001**
(dyne.sec.cm^-5^)	max.increase	**904.73 **± 376.18	**791.69 **± 464.17	**741.30 **± 528.75	ns	ns

Blood volume, blood pressure (BP), heart rate, stroke volume and peripheral resistance fluctuations during dialysis using acetate dialysate (A) vs. citrate dialysate with (C+) or without (C) calcium supplementation.

**Figure 9:**
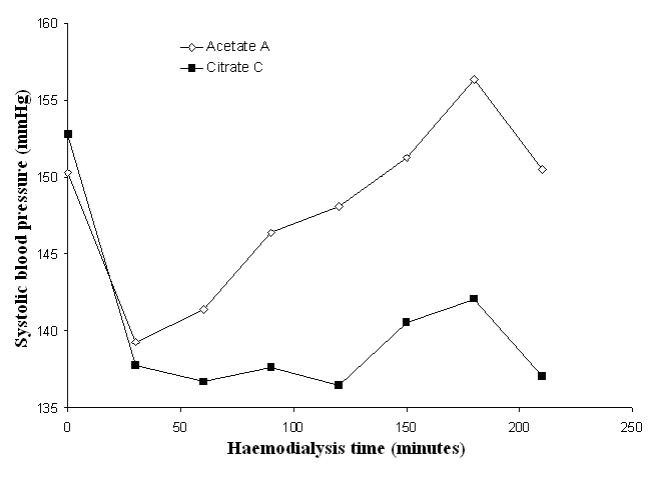
**Systolic blood pressure in hypertensive patients**. Systolic blood pressure as a function of the haemodialysis time using, respectively, acetate (A) (empty diamonds) and calcium uncorrected citrate (C) (black squares) dialysate in the subgroup of patients with an intradialytic systolic blood pressure increase during dialysis of more than 15 mmHg and at least one value of more than 150 mmHg (*n *= 8).

In analysing post-hoc the subgroup of patients with an intradialytic systolic blood pressure increase of more than 15 mmHg and at least one value of more than 150 mmHg (n = 8), we found that pressure values in citrate patients were even lower (-7.8 mmHg; P < 0.05) (see Figure 9 for details).

### Acid-base status and electrolyte changes

The use of citrate dialysate resulted in a significant increase in steady state pre-dialysis bicarbonate levels and a decrease in post-dialysis phosphate levels (+0.95 and -0.13 mmol/L; *p *< 0.01 and *p *< 0.001 respectively). The potassium balance was not influenced significantly (see Table 4 for details).

**Table 4:** Acid-base status, electrolyte changes and dialysis efficiency.

		**Acetate (A)**	**Citrate (C)**	**P(A vs.C)**
		
**Ultrafiltered volume **(L)		**1.88 **± 0.98	**1.85 **± 0.96	ns
**Dialyses with need of staff intervention **(%)		**4**	**3**	ns
**Bicarbonate **(mmol/l)	pre-dialysis	**20.6 **± 2.5	**21.5 **± 2.8	**< 0.01**
	post-dialysis	**25.8 **± 1.9	**24.3 **± 2.4	**< 0.001**
**pH**, whole blood	pre-dialysis	**7.33 **± 0.04	**7.33 **± 0.04	ns
	post-dialysis	**7.43 **± 0.03	**7.41 **± 0.03	**< 0.001**
**Phosphate **(mmol/l)	pre-dialysis	**1.58 **± 0.39	**1.54 **± 0.37	ns
	post-dialysis	**0.62 **± 0.15	**0.49 **± 0.13	**< 0.001**
**Potassium**, serum (mmol/l)	pre-dialysis	**4.74 **± 0.72	**4.78 **± 0.66	ns
	post-dialysis	**3.68 **± 0.26	**3.70 **± 0.20	ns
**Creatinine **(umol/l)	pre-dialysis	**494 **± 147	**480 **± 166	ns
	post-dialysis	**189 **± 60	**182 **± 68	ns
**BUN **mmol/l	pre-dialysis	**21.7 **± 6.5	**19.8 **± 6.6	**< 0.05**
	post-dialysis	**6.0 **± 2.8	**5.6 **± 2.4	ns
**Kt/V**	**1.52 **± 0.37	**1.53 **± 0.31	ns	

Ultrafiltered volume, % of dialyses requiring staff intervention, bicarbonate, phosphate, potassium, pH in whole blood, creatinine, BUN at the beginning and at the end of the dialysis sessions and Kt/V using acetate dialysate (A) vs. citrate (C) dialysate without calcium supplementation.

Unlike the absence of changes in the pre-dialysis ionised calcium levels, those measured post-dialysis were significantly lower in patients receiving citrate dialysate (- 0.08 mmol/L; *p *< 0.001). In the subgroup of patients treated with 1.25 mmol calcium/L in the dialysis fluid (n = 6), an asymptomatic post-dialysis hypocalcaemia was detected (0.99 vs. 1.09 mmol/L post-dialysis ionised calcium/L; *p *< 0.01). Supplementing the citrate dialysate with 0.25 mmol calium/L (C+) completely corrected the difference in post-dialysis ionised calcium previously observed between the two dialysis schedules (see Table 5 for details).

**Table 5:** Calcium balance.

		Acetate (A)	Citrate (C)	Citrate (C+)	P(Avs.C)	P(Avs.C+)
**Calcium**, serum, ionised (mmol/l)	pre-dialysis	**1.19 **± 0.09	**1.20 **± 0.07	**1.23 **± 0.08	ns	**< 0.01**
	post-dialysis	**1.22 **± 0.10	**1.14 **± 0.10	**1.31 **± 0.10	**< 0.001**	**< 0.001**
**Patients with dialysate calcium concentration of 1.25 mmol/l**						
**Calcium**, serum, ionised (mmol/l)	pre-dialysis	**1.14 **± 0.04	**1.16 **± 0.04	**1.16 **± 0.06	ns	ns
	post-dialysis	**1.09 **± 0.03	**0.99 **± 0.02	**1.13 **± 0.07	**< 0.01**	ns
**Patients with dialysate calcium concentration of 1.50 mmol/l**						
**Calcium**, serum, ionised (mmol/l)	pre-dialysis	**1.19 **± 0.10	**1.21 **± 0.08	**1.24 **± 0.08	ns	**< 0.05**
	post-dialysis	**1.27 **± 0.06	**1.19 **± 0.05	**1.34 **± 0.05	**< 0.001**	**< 0.001**

Serum ionised calcium at the beginning and at the end of the dialysis sessions using acetate dialysate (A) vs. citrate dialysate with (C+) or without (C) calcium supplementation.

The value determined for the calcium gap defined as (post-predialysis Δ total calcium) – (post-predialysis Δ ionized calcium), was proportional to unmetabolised citrate accumulation and allowed us to classify patients as rapid or slow metabolisers (post-hoc analysis). In the slow-metabolizer subgroup (n = 9), there were significantly more patients with a net ultrafiltration during dialysis that was > 2L (64 vs. 8%; *p *< 0.05); a similar trend, although nonsignificant, was noted for diabetics (67 vs. 31%). The proportion of rapid vs. slow metabolisers was not influenced by other patient characteristics (gender, weight) or haemodialysis parameters (dialyser effective surface area and dialysis duration).

### Dialysis efficiency

As also observed in previous studies, the use of citrate was associated with a significant decrease in steady state pre-dialysis BUN (-1.8 mmol/L; *p *< 0.05). There was a similar albeit nonsignificant trend for post-dialysis BUN (- 0.4 mmol/L) and consequently for Kt/V (+0.02) (see Table 4 for details).

As stated above, phosphate removal was significantly increased (comparing acetate with citrate: -0.13 mmol/L in post-dialysis phosphate concentration; *p *< 0.001).

### Coagulation parameters

Measurements of thrombin-antithrombin complexes (TAT) and prothrombin fragments 1+2 (F1+2) indicated a significant systemic activation of the coagulation cascade during dialysis that was not influenced by the choice of dialysate (see Table 6 for details).

**Table 6:** Coagulation parameters.

		Acetate (A)	Citrate (C)	P(A vs.C)	P (pre-post)
**Whole group**					
**TAT**, ug/l	pre-dialysis	**4.34 **± 1.90	**4.52 **± 3.29	ns	**A < 0.05**
	post-dialysis	**7.05 **± 6.14	**7.76 **± 8.97	ns	**C **ns
**F1+2, **pmol/l	pre-dialysis	**275 **± 200	**252 **± 183	ns	**A **ns
	post-dialysis	**277 **± 230	**278 **± 281	ns	**C **ns
**Patients with a more pronounced coagulation activation (doubling or more of the pre-dialysis TAT); n = 7; post-hoc analysis**					
**TAT**, ug/l	pre-dialysis	**5.22 **± 2.22	**4.28 **± 1.19	ns	**A < 0.01**
	post-dialysis	**14.9 **± 6.78	**17.0 **± 13.34	ns	**C < 0.01**
**F1+2, **pmol/l	pre-dialysis	**403 **± 181	**374 **± 228	ns	**A < 0.05**
	post-dialysis	**560 **± 194	**577 **± 391	ns	**C < 0.05**

Pre- and post-dialysis thrombin-antithrombin complexes (TAT) and prothrombin fragments 1+2 (F1+2) using acetate dialysate (A) vs. citrate (C) dialysate without calcium supplementation.

### Citrate tolerance

The need for intervention by the medical staff was the same with the two dialysis schedules (see Table 4 for details). The subjective tolerance for citrate and acetate was also similar, with a trend favouring citrate (80% of the patients reported no changes while 16% noted an improvement and 4% a worsening with citrate instead of acetate). Adverse events or significant side effects were not reported.

## Discussion

### Haemodynamic consequences of dialysis fluids containing citrate instead of acetate

The use of citrate in dialysis fluids was associated with significant haemodynamic changes during haemodialysis. Specifically, we found a mean decrease in systolic and diastolic blood pressures (4.3 and 1.6 mmHg respectively) mediated by a reduction in peripheral resistances (-51 dyne.sec.cm^-5^) and incompletely compensated by a nonsignificant increase in stroke volume (5.0 ml) (Table 2). The changes in peripheral resistances were independent of the effect of citrate on calcium balance, as confirmed by the use of a dialysate supplemented with the amount of calcium necessary to compensate for its binding to citrate (0.25 mmol/L) (Table 2). Even if the citrate modality was associated with both lower mean systolic and diastolic BP and mean peripheral resistances, analysis of the extremes of fluctuations of the latter parameter suggested that citrate use improved systemic haemodynamic stability. In fact, despite the contrasting behaviour of BP, the maximum decrease and increase in peripheral resistances was more pronounced with acetate use (maximum decrease 974 vs. 485 and maximum increase 905 vs. 792 dyne.sec.cm^-5^) (Table 3).

Furthermore, a post-hoc analysis showed that the citrate-containing dialysate resulted in a more pronounced reduction in blood pressure in the subgroup of patients with an intradialytic hypertension (Figure 9).

### Acid-base status and electrolyte changes

As expected, citrate dialysis was associated with an increase in steady-state pre-dialysis bicarbonate. The consequences of citrate on bicarbonataemia may be explained by the incomplete conversion of citrate into bicarbonate during the dialysis session, as confirmed by the increased gap between total and ionised calcium (calcium gap), reflecting the hepatic and muscular metabolism of citrate that occurred in part after the end of the dialysis session. Interestingly it was also observed that peripheral perfusion and muscle mass are probably related to the rate of intra-dialytic citrate removal. This was established by classifying patients as rapid or slow metabolisers according to their calcium gap (proportional to unmetabolised citrate). Among the slow metabolisers, the percentage of patients with a net ultrafiltration during dialysis > 2L was significantly higher (64 vs. 8%) while a similar, although nonsignificant trend was seen in diabetics (67 vs. 31%) (see Table 6 for details). The accumulation of citrate in these patients could in fact be explained by a lower muscle mass or a less efficient muscle perfusion related to vasoconstriction.

Since the dialysis sessions lasted no more than 4 hours and the calcium gap did not correlate with dialysis duration, the amount of unmetabolised citrate resulting from longer haemodialyses could not be determined in the present study.

While there were no changes in the pre-dialysis ionised calcium, post-dialysis values were significantly lower in the group using the citrate dialysate (1.22 vs. 1.14 mnmol/L; -7%). As expected, patients treated with a dialysis fluid containing 1.25 mmol calcium/L, were found to have an asymptomatic post-dialysis hypocalcaemia. Supplementation of the dialysate with 0.25 mmol calcium/L completely corrected the difference in post-dialysis calcium concentration between the acetate and citrate modalities (see Table 5 for details). Post-dialysis hypocalcaemia was always asymptomatic and patients spontaneously recovered in the interdialytic phase. Nevertheless, patients treated with a dialysate in which the calcium concentration is 1.25 mmol/L require careful monitoring of the serum calcium when converted to citrate-based dialysis, including, if indicated, an adaptation in the amount of calcium in the dialysate.

### Dialysis efficiency

As shown in previous studies, the use of citrate was associated with an increased removal of phosphate and urea. In contrast to Ahmad's study [22], in which a significant difference was reported, we observed only a trend favouring an increase in Kt/V; the absence of significance/statistical power may have been due to the use of single-use dialysers (see Table 4 for details).

In addition, since a correction of acidosis improves catabolism [27], the lower pre-dialysis BUN might be explained by a reduced generation of urea, resulting from the improved acid-base status obtained using citrate.

### Coagulation parameters

Thrombin-antithrombin complexes and prothrombin fragments 1+2 are very sensitive markers of systemic coagulation activation during haemodialysis [9]. The absence of significant changes in their concentrations between the two dialysis schedules leads to the conclusion that systemic coagulation activation was not influenced by dialysate composition. This confirms that the effect of citrate is limited to the dialyser and that the inhibition of the coagulation cascade taking place in the extracorporeal circuit is incomplete with either method. Since in the present study the extracorporeal circuit was not evaluated for clotting and the heparin dose was not down-titrated, the clinical relevance of the intradialyser coagulation inhibition related to the use of citrate cannot be conclusively stated.

### Citrate tolerance

Intervention by the medical staff was the same in the citrate group as in the acetate group. The responses to a patient questionnaire assessing subjective tolerance of the dialysis sessions indicated a nonsignificant trend in favour of citrate.

## Conclusion

The use of citrate rather than acetate as a dialysate in bicarbonate haemodialysis decreases peripheral resistances and slightly reduces systolic and diastolic blood pressures. Nonetheless, both the analysis of maximum fluctuations in peripheral resistances during dialysis and data describing subjective tolerance suggest a trend towards improved haemodynamic stability for patients on the citrate schedule. Interestingly, patients developing intradialytic hypertension experienced a larger reduction in blood pressure. Even with single-use dialysers, citrate resulted in better urea and phosphate removal, probably by preventing clotting of the dialyser fibres. To maintain the same intradialytic calcium balance following the switch to citrate, the calcium concentration of the dialysate should be increased of about 0.25 mmol/L. However, considering that steady-state pre-dialysis calcium was not significantly modified by the use of citrate, the need for calcium supplementation of the dialysate, particularly in patients treated with a 1.25 mmol calcium/L regimen, should be individually determined. In the citrate modality, a systemic effect on the coagulation cascade, as evaluated by specific markers, was not detected. This was probably associated with reduced intra-dialyser coagulation activation. Therefore, even if the use of citrate dialysate is recommended, in order to reduce the need for anticoagulants in patients at risk of bleeding, this aspect needs further investigation.

The positive impact of the citrate dialysate on dialyser efficiency, on acid-base status and the haemodynamic pattern as well as the subjective tolerance together suggest that the substitution of citrate for acetate in dialysis fluids improves haemodialysis in selected patients.

## Competing interests

The authors declare that they have no competing interests.

## Authors' contributions

LG was involved in the study design, sample collection, analysis and interpretation of the data and writing of the report; BL participated in the sample collection, analysis and interpretation of the data and in the writing of the paper; CM, LA and MB helped formulate the study design, the data analysis strategy and contributed to the writing of the paper. All authors read and approved the final manuscript.

## Pre-publication history

The pre-publication history for this paper can be accessed here:

http://www.biomedcentral.com/1471-2369/10/7/prepub
